# Development and Application of a High-Precision Portable Digital Compass System for Improving Combined Navigation Performance

**DOI:** 10.3390/s24082547

**Published:** 2024-04-16

**Authors:** Songhao Zhang, Min Cui, Peng Zhang

**Affiliations:** School of Instrument and Electronics, North University of China, Taiyuan 030051, China; song1997hao2021@163.com (S.Z.); 20051588@nuc.edu.cn (M.C.)

**Keywords:** digital compass, tunnel magnetoresistance effect, combined navigation system, precision, stability

## Abstract

There are not many high-precision, portable digital compass solutions available right now that can enhance combined navigation systems’ overall functionality. Additionally, there is a dearth of writing about these products. This is why a tunnel magnetoresistance (TMR) sensor-based high-precision portable digital compass system is designed. First, the least-squares method is used to compensate for compass inaccuracy once the ellipsoid fitting method has corrected manufacturing and installation errors in the digital compass system. Second, the digital compass’s direction angle data is utilized to offset the combined navigation system’s mistake. The final objective is to create a high-performing portable TMR digital compass system that will enhance the accuracy and stability of the combined navigation system (abbreviated as CNS). According to the experimental results, the digital compass’s azimuth accuracy was 4.1824° before error compensation and 0.4580° after it was applied. The combined navigation system’s path is now more accurate overall and is closer to the reference route than it was before the digital compass was added. Furthermore, compared to the combined navigation route without the digital compass, the combined navigation route with the digital compass included is more stable while traveling through the tunnel. It is evident that the digital compass system’s design can raise the integrated navigation system’s accuracy and stability. The integrated navigation system’s overall performance may be somewhat enhanced by this approach.

## 1. Introduction

As navigation technology grows in popularity, many nations have made significant financial, material, and labor investments in high-precision navigation. All nations are concentrating their attention on high-precision navigation techniques. Several nations have designated research hotspots for the development of highly precise and broadly applicable navigation technologies [1].

A digital compass, sometimes referred to as an electronic compass, measures azimuth by detecting geomagnetic signals. It can be used to calibrate the accumulated mistakes of the angular velocity sensor (gyroscope), giving the navigation system steady and accurate azimuth information. This is due to its low cost, high flexibility, and low tendency to accumulate errors. Many industries, including directional navigation, precise guidance, aviation control, and navigation surveying, heavily rely on this technology. Digital compass technology has attracted strong attention from countries around the world [2].

At present, the sensors used in digital compasses on the international market are developed based on the magnetoresistance effect. The magnetoresistance effect is further subdivided into four types. The first is the Hall (abbreviation: HALL) effect. The second type is the anisotropic magnetoresistance (abbreviation: AMR) effect. The third type is the giant magnetoresistance (abbreviation: GMR) effect. The fourth type is the tunnel magnetoresistance (abbreviation: TMR) effect. The comparison of their advantages and disadvantages is shown in Table 1.

A combined navigation system (CNS) is a type of navigational system that combines various sensors and technologies toward providing increased accuracy, reliability, and durability while providing location, velocity, and orientation data. These kinds of systems often combine different kinds of sensors to reduce the impact of individual limitations and provide navigation solutions that work consistently in a range of environmental conditions.

Global Navigation Satellite System (GNSS)/Inertial Navigation System (INS) combined navigation is currently the most widely utilized combined navigation technique used worldwide. This system combines positioning information provided by satellites. Simultaneously, INS relies on inertial device measurements to provide more accurate and reliable navigation solutions. Therefore, this navigation method is widely used in fields such as navigation, land vehicle navigation, and unmanned systems (such as unmanned aerial vehicles and autonomous vehicles). It is particularly important in scenarios that require high navigation precision and real-time performance [3,4].

However, GNSS/INS CNS also has some disadvantages. When GNSS passes through obstructed buildings such as tunnels and bridges, satellite signals will be blocked, which will lead to increased navigation errors and reduced precision [5]. For INS, as time accumulates, cumulative errors will occur. The longer the time, the greater the cumulative error, which requires timely correction by other navigation equipment [6,7,8].

Building upon the existing research landscape, the aim of this paper is to create a highly accurate and portable digital compass system, employing it to enhance the precision and stability of the GNSS/INS CNS.

This paper mainly accomplishes two tasks. The first is to use a compensation method based on the least-squares method to develop a high-precision portable digital compass system and test its accuracy, volume, and weight. The second is to carry out practical testing of the developed digital compass system in the vehicle-mounted GNSS/INS-integrated navigation system. The purpose is to enhance the precision and stability of the combined navigation system. After a sequence of experiments, it was observed that the created high-precision, portable digital compass system possesses the benefits of precision, a compact size, and light weight, contributing to the enhancement of precision and stability in the combined navigation system. Through this research, a digital compass system is designed that is highly accurate, easy to carry, and can improve the overall performance of the integrated navigation system. The digital compass system designed in this article fills a gap in the market and makes up for the lack of relevant information on such products in the current literature. It has played a certain role in promoting the development of integrated navigation systems.

## 2. Design of High-Precision Portable Digital Compass System

### 2.1. Design of Overall System Plan

**Figure 1:**
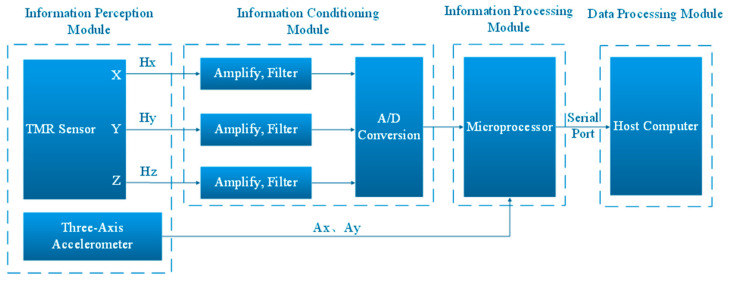
Block diagram of the overall scheme design of a high-precision portable digital compass system.

There are four modules in the overall system plan. The information perception module is the first. Signal conditioning makes up the second module. The information processing module is the third one. The data output module is the fourth. Figure 1 illustrates the overall design block diagram of the high-precision portable digital compass system. First, three TMR sensors sense magnetic signals in their respective axes. The sensed information is then passed to amplification and filtering circuits [9]. After completion, the signal that meets the requirements is sent to an Analog-to-Digital Converter (abbreviation: ADC) and performs analog-to-digital conversion. Finally, the digital signal is transmitted to the microprocessor. At the same time, the acceleration information obtained by the accelerometer is also transmitted to the microprocessor. Inside the microprocessor, a series of steps such as attitude information calculation and correction are performed. Following the information processing by the microprocessor, the ultimate outcome is sent to the host computer through the serial port for immediate presentation [10].

### 2.2. Hardware Design

#### 2.2.1. Information Sensing and Conditioning Circuit for Magnetic Fields

**Figure 2:**
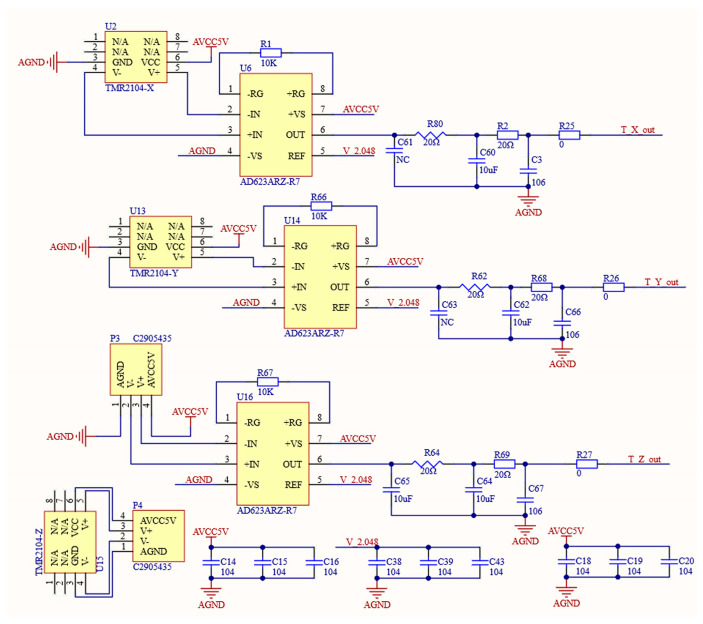
Information sensing and conditioning circuit for magnetic fields.

The magnetic field sensing end of the system uses the TMR2104 model magnetic sensor to realize the magnetic sensing function. The following factors were the primary motivators for selecting this sensor. Firstly, at 3.1 mV/V/Oe, the device’s sensitivity is high. Furthermore, the apparatus demonstrates remarkable stability in relation to temperature [11,12,13]. Since the magnetic sensor is a Wheatstone bridge output type and does not have buffering and strong driving capabilities, an instrumentation amplifier is introduced at its back end specifically for weak driving sensing signals. The goal of this design is to effectively amplify the signal. After researching instrumentation amplifiers on the market, we selected the AD623ARZ-R7 because of its low noise, high precision, and high input impedance. Additionally, its amplification can be adjusted via external resistors. Subsequently, a second-order passive filter circuit was connected to further optimize the signal [14]. Figure 2 depicts the information sensing and conditioning circuit for magnetic fields.

#### 2.2.2. Circuit for Acceleration Information Sensing

**Figure 3:**
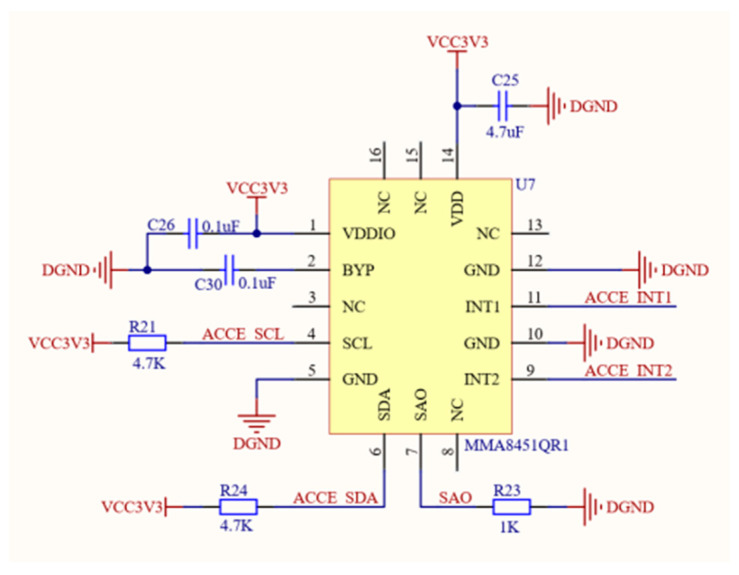
Circuit for acceleration information sensing.

As a key component for correcting geomagnetic information, the precision of the accelerometer also needs to be ensured [15]. After comprehensively considering factors such as performance and volume, we selected the MMA8451QR1 three-axis accelerometer. This accelerometer was selected for three key reasons. First off, MMA8451QR1 has a 14-bit output number. Second, this accelerometer has a low noise level; its noise value, 99 μg/√Hz, falls into this category. Therefore, its accuracy is very high. Figure 3 displays a circuit for acceleration information sensing.

#### 2.2.3. Circuit for Analog-to-Digital Conversion

**Figure 4:**
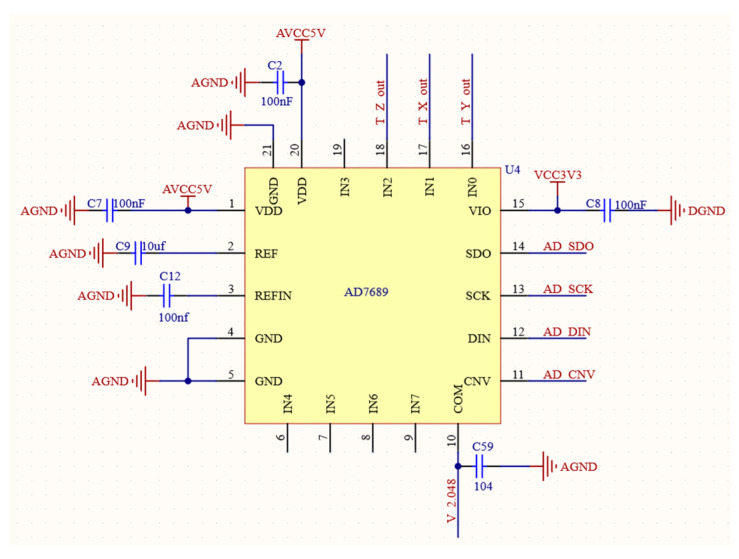
Circuit for analog-to-digital conversion.

The digital compass system is three-dimensional, meaning it has three axes. Therefore, in order to convert the three-axis geomagnetic signal from analog to digital, there must be a minimum of three Analog-to-Digital Converter (abbreviation: ADC) channels [16,17]. To fulfill this requirement, the AD7689ACPZ model ADC is selected as the core of this part. The circuit for analog-to-digital conversion is presented in Figure 4. This ADC is an eight-channel successive-approximation register. It has all the components required for a multi-channel, low-power data acquisition system and uses a basic SPI interface to write configuration registers and receive conversion results. Because of this, the AD7689ACPZ fully meets the comprehensive channel count and related performance requirements.

#### 2.2.4. Circuit for Information Processing

**Figure 5:**
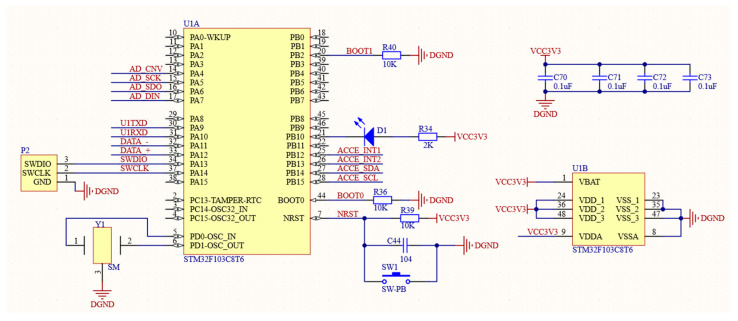
Circuit for information processing diagram.

The STM32F103C8T6 model is the specific microcontroller model utilized, which belongs to the STM32 series. In addition, it also has the following features. Firstly, it has excellent fast processing capabilities, with a core frequency of up to 72 MHz. Secondly, it has strong peripheral support, including an Analog-to-Digital Converter (abbreviation: ADC), a Digital-to-Analog Converter (abbreviation: DAC), an Inter-Integrated Circuit (abbreviation: I2C), a Serial Peripheral Interface (abbreviation: SPI), a Universal Synchronous Asynchronous Receiver Transmitter (abbreviation: USART), and other commonly used peripherals to meet various application needs. Third, it has strong scalability and can connect more peripherals or access external memory. Finally, power consumption is low. STM32F103C8T6 provides a variety of low-power modes, which can effectively reduce power consumption while ensuring performance. In this system, STM32F103C8T6 plays the following key roles: First, it performs attitude calculation of magnetic signals. The second is to use gravity field information to perform rotor error correction. The third is to communicate in real time with the host computer [17]. The circuit for the information processing diagram is shown in Figure 5.

#### 2.2.5. Circuit for Serial Communication

**Figure 6:**
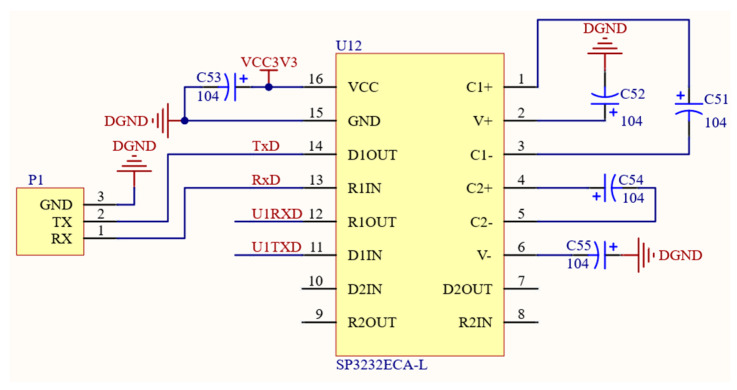
Serial communication circuit diagram.

Within the system, there exists a discrepancy between the output level post-analog-to-digital conversion and the microcontroller’s input/output (abbreviation: I/O) port level. To address this issue, a level conversion is necessary [18]. Figure 6 illustrates the circuit diagram for serial communication. The chip model SP3232ECA-L was selected to meet this demand. In addition, the serial communication function of RS232 is also covered in this part.

#### 2.2.6. Electrical Power Circuit

In the system circuit, the analog part requires a supply voltage of 5 V. The digital part requires a supply voltage of 3.3 V. For this reason, the Micro USB interface is selected as the power supply interface of the overall system. The interface outputs a 5 V voltage to power the analog part. The buck circuit converts the input 5 V voltage into 3.3 V to power the digital part.

Adhering to the design principle of “lower voltage difference, choose buck, larger voltage difference, boost” in the circuit design, the external power supply of the system is 5 V, and a buck module is designed inside the circuit to reduce the 5 V voltage to 3.3 V. This ensures power supply everywhere. The buck chip selection is ADP1710AUJZ-3.3-R7. Its noise is 40 μVRMS, which is low noise. The load precision is ±2%.

**Figure 7:**
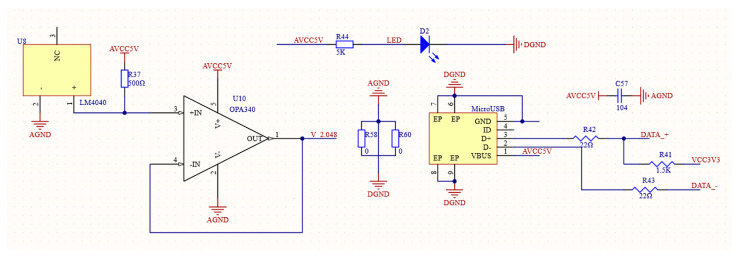
Circuit diagram of electrical power.

The AD7689ACPZ offers three choices for its reference voltage: external, internal, or a combination of external and internal buffers. Figure 7 illustrates the schematic diagram of the electrical power circuit. To guarantee the digital compass’s portability, an internal reference voltage source solution has been chosen. The voltage reference chip LM4040 is selected here. Its precision is 2.048 mV (±0.1%×2.048 v=2.048 mV), which meets the voltage requirements. Since the output voltage of the AD7689ACPZ is 4.096 V, the common-mode voltage of the instrumentation amplifier AD623ARZ-R7 is chosen to be half the ADC voltage value, which is 2.048 V. The LM4040 chip can just provide the common-mode voltage of AD623ARZ-R7. The LM4040 chip has a typical noise level of 35 μVrms, a precision level of ±0.1%, superior performance, and simple circuit connections. To enhance the driving capability of LM4040, a voltage follower OPA340 is added to the output end.

In the hardware design, except for the power circuit, which is well known, all other circuits are developed by us.

### 2.3. Software Design

**Figure 8:**
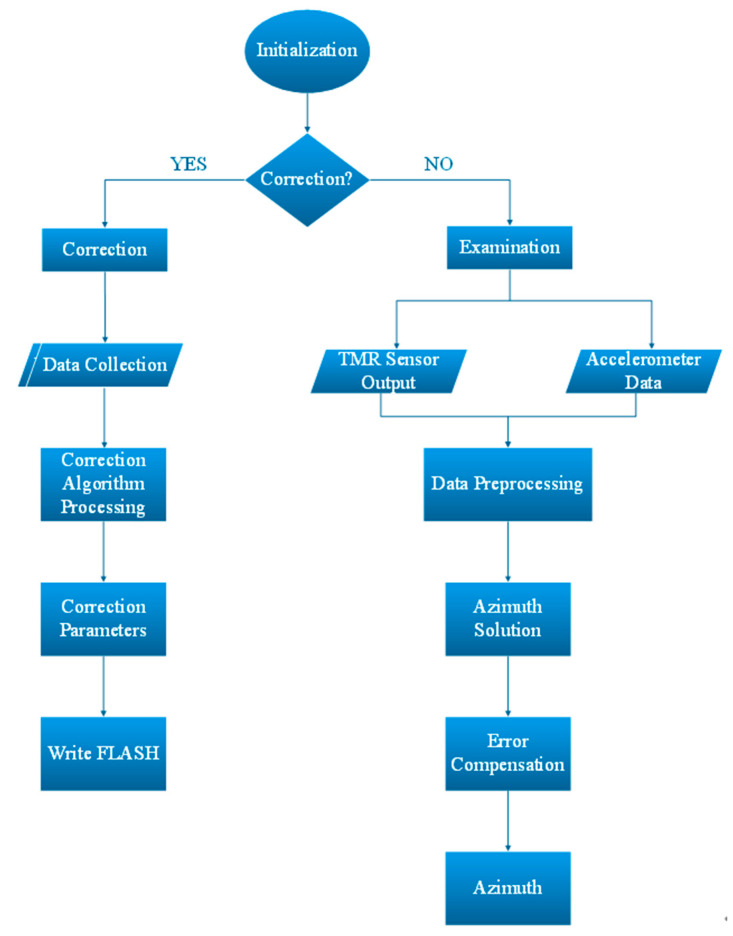
Overall flow chart for software design.

Using the Keil 5 development platform as the foundation for hardware implementation, C programming concepts are used to carry out programming. The software’s overall design process is illustrated in Figure 8. It mainly implements the following core functions: Firstly, it completes the collection and control functions of the sensor data. Secondly, the correction, compensation, and calculation functions of the heading angle are realized. Finally, the real-time display memory function of the data is completed. Software development is mainly divided into four parts: data acquisition, azimuth angle solution, error parameter acquisition and correction, and error compensation. Specific operations include data collection and processing, error compensation, azimuth angle calculation, etc.

The following six sub-modules comprise software development. The system startup module is the first. The module for collecting data is the second. The data calibration module comes in third. The serial communication module is the fourth [19]. The module for calculating azimuth angles is the fifth. The module for displaying and storing real-time data is the sixth. The discussion of each sub-module follows.

#### 2.3.1. Module of Initialization

Upon the system startup, the microcontroller initiates and sets up the on-chip peripherals, including general-purpose input/output (abbreviation: GPIO), clock, timer, USART, and others, that need to be used. In addition, the ADC and accelerometer need to be functionally initialized before use [20].

The initialization process for the AD7689ACPZ analog-to-digital converter involves configuring the SPI bus interface of the controller responsible for communication with the converter. The ADC itself does not need to be initialized and configured. In actual use, its input signal type, reference voltage, channel selection, channel sequencer, and other working methods can be configured in real time during the data reading operation.

In addition to the communication interface configuration of the controller, the initialization of the accelerometer MMA8451QR1 requires related operations such as its working mode and range selection. The main ones are while in the Standby state, select ±2 g as the range, and the data output frequency is 200 Hz. The automatic wake-up sampling frequency is 50 Hz.

#### 2.3.2. Module for Collecting Data

AD7689ACPZ obtains the geomagnetic field information after conditioning the circuit, performs an analog-to-digital conversion, and communicates with the microprocessor through SPI. When reading channel data, you need to first configure the specific working mode of AD7689ACPZ. During practical application, set the input channel to bipolar mode, utilizing the voltage value “$\frac{V_{ref}}{2}$” as the reference voltage. In addition to this, configure the AD7689ACPZ’s reference voltage source as internal, disable the channel sequencer, and set the CFG configuration word to prevent data readback after reading. The above configuration can be achieved by writing the CFG configuration word to AD7689ACPZ. AD7689ACPZ only reads the data of one channel at a time. Therefore, reading the output of the three-dimensional magnetic sensor requires writing the CFG configuration word three times.

The accelerometer MMA8451QR1 three-axis acceleration information can be directly output digitally and communicates with the microcontroller through IIC. After the MMA8451QR1 is initialized, activate the accelerometer to enter the normal working mode and determine whether the ZYXDR_MASK flag is 1. If it is true, it means that the data of the three channels of the MMA8451QR1 are ready on the bus. Read and write the data of each channel in turn. Since the IIC bus can only output 8-bit data at a time.

The data of one channel need to be divided into high 8 bits and low 8 bits and read twice. The MMA8451QR1 only supports 14-bit output, so the first two datapoints of the lower 8 bits are 0, which has no practical significance.

#### 2.3.3. Module for Data Calibration

Due to the inevitable manufacturing and installation errors of magnetic sensors and accelerometers in the digital compass design process, the digital compass needs to be calibrated when used for the first time. The obtained calibration parameters can be directly used for the current test or can be stored. Flashing for use in the next test; since the environment, location, time, etc. of each use are different, it is recommended to test after each power-on [21].

During use, strong magnetic fields may interfere, resulting in abnormal azimuth angle test results. At this time, the impact of external magnetic fields on test interference can also be reduced through correction.

The calibration mode can be started through the calibration button in the host computer software. After the calibration is started, the digital compass needs to be manually operated so that its posture occupies the entire space of the entire ellipsoid as much as possible. The data will be automatically analyzed to determine whether the amount of data is sufficient. That is to say, cease the data gathering if the amount is adequate. The acquired data are solved by the ellipsoid fitting algorithm to obtain the zero bias, scale factor, non-orthogonal angle, and other parameters of the sensor and accelerometer. The parameters are stored in Flash for use in azimuth angle calculation.

#### 2.3.4. Module for Serial Communication

For the ease of the data analysis, the system transmits gathered magnetic field and acceleration information, along with calculated azimuth and attitude angles, to the host computer via the serial port. As a result, the first stage in the software design of the serial communication module is to configure the microcontroller’s serial port peripherals, including the baud rate, data bits, check bits, and other parameters [22]. Table 2 displays the designed data transmission protocol that aims to guarantee the quality of data delivery.

The protocol includes regulations on the header and tail of data packets and a unified arrangement of the order of data transmission.

The header consists of two headers, data type, and data length. The data length indicates the size of the data to transmit. The data include the axial magnetic sensor output, three-axis accelerometer output, and computed azimuth, roll, and pitch angles, totaling nine items.

#### 2.3.5. Module for Calculating Attitude Angle

Since the TMR sensor and accelerometer itself have manufacturing and installation errors and are more susceptible to magnetic field interference from the surrounding environment, there is a large error in directly solving the azimuth angle. Therefore, the data need to be corrected first, the correction parameters are extracted from FLASH, and the corrected data is solved.

The resolved azimuth data will use a lot of resources during serial data transmission because it is floating-point data. Therefore, multiplying the data to be sent by 10,000 not only ensures the precision of the data but also makes the data transmission convenient and fast.

#### 2.3.6. Module for Real-Time Data Display and Storage

The data collected in the program are sent through the serial port. After the data are transmitted to the host computer, the real-time display and storage functions of the data are realized: displays of the azimuth angle, attitude angle, and real-time test values of the TMR sensor and accelerometer.

### 2.4. Construction of Error Compensation Algorithm

#### 2.4.1. Building the Compass Error Model

Although digital compass errors cover quadrant errors, etc., the most important error affecting the precision of digital compasses is compass error because the system is composed of both soft magnetic materials and hard magnetic materials [23,24]. Therefore, the error model of the total error of the system is

(1) $$∆\Phi =∆\Phi_{1}+\Delta \Phi_{2}$$

In the formula, the deviation induced by soft magnetic materials is $\Delta \Phi_{2}$ and the unit is degrees (°); the deviation induced by hard magnetic materials is $∆\Phi_{1}$ and the unit is degrees (°); and the overall deviation of the system is ∆Φ and the unit is degrees (°).

For hard magnetic interference, because the magnetic sensor has a small size, it can be regarded as having a uniformly distributed magnetic field of hard magnetic material around the sensor. Simultaneously, being affixed to the carrier, the combined magnetic field produced by the hard magnetic material remains constant across all three axes regardless of any alterations in the carrier’s orientation. The error generated thereby changes with the direction angle 0 to 2π. The created curve now exhibits a semicircular deviation, meaning that it is approaching the shape of a sine. The following formula can be used to define this finding:

(2) $$∆\Phi_{1}=B\sin\Phi +C\cos\Phi ,$$

In the formula, the output azimuth angle of the digital compass system before compensating is Φ in degrees (°); B and C are both error compensation coefficients.

For soft magnetic interference, the soft magnetic material does not inherently produce a magnetic field. When it is magnetized, it affects the magnetic field. Under this influence, changes in the external magnetic field affect the error. It is divided into two parts: one is a circular error and the second is a quadrant error. It can be presented in the following ways:

(3) $$∆\Phi_{2}=A+E\cos2\Phi +D\sin2\Phi ,$$

In the formula, the error compensation coefficients are A, D, and E; the output azimuth angle before compensation is Φ and the unit is degrees (°).

Drawing from the preceding information, the error model can be formulated as follows:

(4) ∆Φ=A+Bsin⁡Φ+Ccos⁡Φ+Dsin⁡2Φ+Ecos⁡2Φ

The relationship between the post-compensation azimuth angle, the pre-compensation output azimuth angle, and the total system error can be analyzed according to the following formula:

(5) $$\Phi_{C}=\Phi -∆\Phi ,$$

In the formula, the output azimuth angle of the digital compass system before compensating is Φ in degrees (°); ∆Φ is the total compass in degrees (°); and the actual azimuth angle after compensating is $\Phi_{C}$ in degrees (°).

#### 2.4.2. Process of the Least-Squares Algorithm

To enhance precision even further, the decision is made to employ the least-squares method for computing the error compensation coefficient. For this algorithm, the minimum sum of error squares is the core [25,26,27,28,29,30,31]. When the range is 0°~360°, 15° is used as an interval, and a total of 24 test points are obtained. Based on this, 24 sets of data can be obtained by conducting experiments. The following formula can be used:

(6) U·M=H,

Among

$$U=\left(\begin{array}{ccccc} 1 & \sin\Phi_{1} & \cos\Phi_{1} & \sin{2\Phi }_{1} & \cos{2\Phi }_{1}\\ 1 & \ddots & & & \vdots \\ 1 & & \ddots & & \vdots \\ \vdots & & & \ddots & \vdots \\ 1 & \sin\Phi_{24} & \cos\Phi_{24} & \sin{2\Phi }_{24} & \cos{2\Phi }_{24} \end{array}\right)$$

$$M=\left(\begin{array}{c} A\\ B\\ C\\ D\\ E \end{array}\right)$$

$$H=\left(\begin{array}{c} \Phi_{1}-0\\ \Phi_{2}-15\\ \vdots \\ \vdots \\ \Phi_{24}-345 \end{array}\right)$$

The calculation of the compensation coefficient matrix M can be completed using the above formulas. Building upon this foundation, the values for A~E are derived. By substituting these values into Equation (4), the accurate calculation formula of the total error of the system can be obtained.

#### 2.4.3. The Combined Navigation Principle (GNSS/INS + COMPASS)

INS can provide position, speed, attitude, and heading completely autonomously, without being subject to external electromagnetic interference. However, due to errors in the gyroscope and meter, errors will accumulate. Therefore, this system cannot operate alone for long periods of time.

To mitigate INS errors, the current most efficient approach involves integrating INS with other navigation devices to establish a CNS. In this regard, the Kalman filter algorithm is employed for handling diverse sensor data. The objective is to assess the errors of the INS and subsequently rectify them.

Although INS can work continuously and effectively provide data, the precision of the INS will gradually decline due to the accumulation of inertial device errors [32,33]. GPS is capable of providing extremely accurate location data; however, the signal is easily jammed and obstructed [34]. Therefore, actual navigation cannot only use the above two methods.

To this end, the developed TMR digital compass is used in conjunction with the vehicle-mounted GNSS/INS-integrated navigation. GNSS is employed for rectifying position and velocity errors in the INS. Simultaneously, the digital compass is utilized to rectify attitude errors in the INS. This approach contributes to enhancing the precision of the entire navigation system. When passing through a tunnel, although the GNSS signal is blocked and the GNSS information is inaccurate, the attitude information in the TMR digital compass is accurate and can continue to provide attitude correction for the INS [35,36,37,38,39,40,41,42,43,44,45,46,47,48,49,50,51,52,53,54,55,56,57,58,59,60,61,62,63].

**Figure 9:**
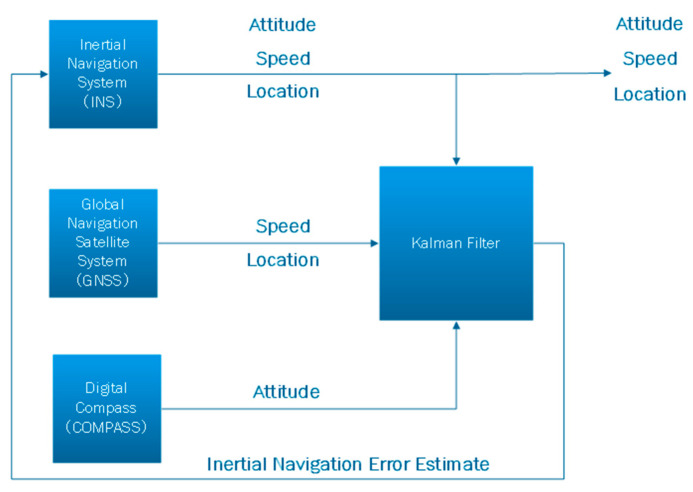
Schematic diagram of COMPASS+GNSS/INS-integrated navigation principle.

Figure 9 illustrates the schematic diagram depicting the integrated navigation principle of COMPASS+GNSS/INS.

## 3. Experimental Platform Construction

### 3.1. Construction of High-Precision Portable TMR Digital Compass Test Platform

**Figure 10:**
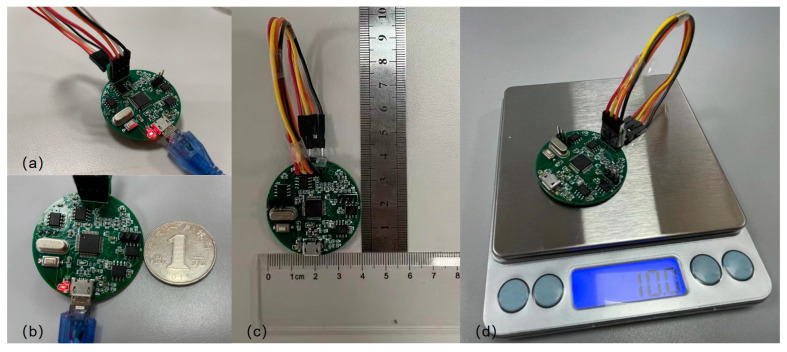
A schematic representation of the test platform for assessing the portability of the digital compass. (**a**) A working diagram of a TMR digital compass; (**b**) a schematic diagram comparing the volume of a TMR digital compass and a CNY 1 coin; (**c**) a TMR-type digital compass volume test chart; and (**d**) a TMR digital compass weight test chart.

**Figure 11:**
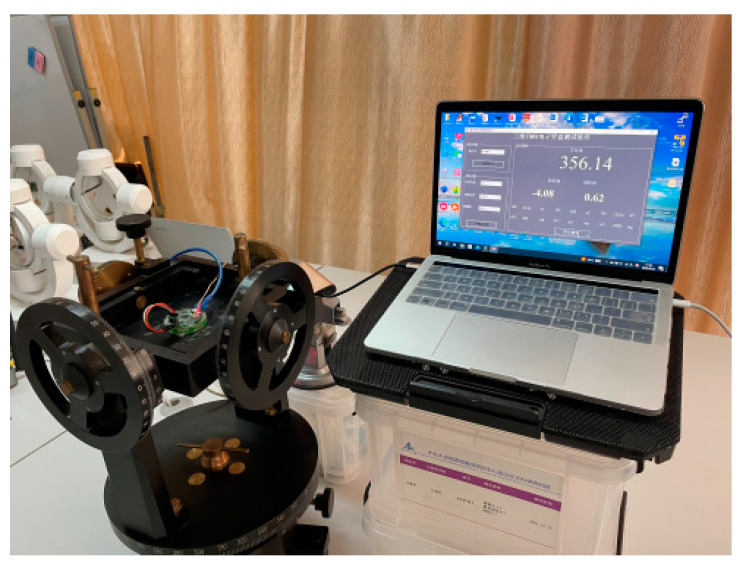
Schematic diagram of digital compass precision test platform.

After the production of the high-precision portable TMR digital compass was completed, the size and weight were inspected using a ruler and an electronic scale, respectively. After the inspection, the diameter of the digital compass was found to be 4 cm, and the weight was 10 g. It is of a small size and light weight type, which fully reflects the portability of the digital compass. Afterwards, the digital compass system prototype was fixed on a non-magnetic turntable for precision-related tests to verify its high-precision performance. Figure 10 displays the schematic diagram of the experimentation platform for assessing the portability of the digital compass. Figure 11 illustrates the schematic diagram of the precision evaluation platform for the digital compass.

The XOY surface of the digital compass prototype is a circular PCB with a diameter of 4 cm. The cross-sectional area of the XOY surface is $4\pi \;{cm}^{2}$ and the thickness is 0.1 cm. The Z-axis loading plate is 1.14 cm long, 1.19 cm wide, and 0.1 cm thick. The total volume is $\left(4\pi +0.13566\right)\;{cm}^{3}$, approximately $1.39166\;{cm}^{3}$, which is $1391.66\;{mm}^{3}$. It is lightweight at 10 g. The azimuth accuracy is 0.4580°. The total cost of a single prototype is CNY 225.97. The maximum output energy consumption is 0.7 W. The digital compass developed in this article can be used to improve the overall performance of the combined navigation system.

The digital compass developed by a Japanese company based on the magnetic impedance change of rapidly quenched amorphous wire is used in the design and manufacturing of some electronic circuits [64,65,66,67,68,69,70]. Compared with Japanese technology, the technology in this article has the advantages of higher accuracy and lower maximum output energy consumption. In addition, a Japanese company has made a digital compass into a sensor through packaging and integration. Although the size of this sensor is smaller than the size of the digital compass system designed in this article, the manufacturing cost is much higher than the digital compass system designed in this article. Compared with Japanese technology, the technology in this article has the advantages of lower manufacturing costs and higher practicability. At the same time, the digital compass sensor produced by a Japanese company is a component and must be used with peripheral circuits. Compared with Japanese technology, the technology in this article has the advantages of being more convenient to use, better portability, and can improve the overall performance of the combined navigation system.

Measurements of the angle dependence of the response of thin-film structures, amorphous ribbon elements, composite wires consisting of a conductive base, and a magnetic covering (GMR or GMI effect) are documented in publications from German, Spanish, and Thai groups [71,72,73,74,75,76,77,78]. The technology used in this article is to develop a digital compass system based on the TMR effect. Compared with the technologies published by German, Spanish, and Thai groups, the technology of this article has the advantages of higher sensitivity, lower energy consumption, smaller sensor size, and higher resolution. This makes the technology in this article more valuable in practical applications.

The digital compass precision performance test steps are as follows:

The first step is to perform ellipsoid fitting correction [79,80,81]. The following are the detailed procedures. Firstly, adjust the non-magnetic turntable to the horizontal position and keep it stable. Then, rotate the prototype so that its different postures cover the three-dimensional space range of the matched ellipsoid as much as possible to complete the matching calibration of the ellipsoid. Use MATLAB 2020A software to calculate the error parameters. The purpose is to achieve the calibration of each sensor.

The second step is to collect data at each location using a non-magnetic turntable and viewing its own reading as the reference angle. Then, start the proficiency test. The specific operation steps are: Firstly, set the non-magnetic turntable’s inner frame to the zero position. Secondly, rotate the inner frame of the non-magnetic turntable with the digital compass system prototype attached horizontally and evenly. For the interval, points are taken at each interval, and data are captured and stored simultaneously.

The error analysis constitutes the third stage. The digital compass system host computer processes the position data read from the non-magnetic turntable at each point, and MATLAB is used to analyze errors both before and after correction.

### 3.2. Construction of Practical Test Platform for Digital Compass in CNS

**Figure 12:**
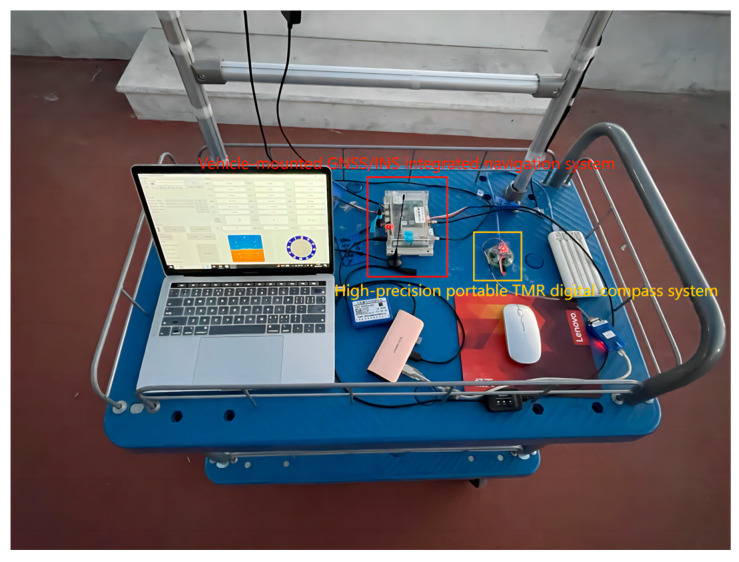
Schematic diagram of the practical test platform for digital compass in CNS.

The practical test of the digital compass in CNS refers to two aspects. One aspect concerns the influence on the total CNS’s precision. The other aspect pertains to the effect on the total CNS’s stability. The experimental site was selected close to the university’s main building. After the high-precision portable TMR digital compass and the vehicle-mounted GNSS/INS CNS were successfully turned on, they were mounted on the car. The specific installation specifications are as follows: The alignment of the digital compass’s three axes should match those of the CNS within the vehicle. Figure 12 illustrates the schematic representation of the practical testing platform for the digital compass within the CNS.

#### 3.2.1. Tests of Precision

**Figure 13:**
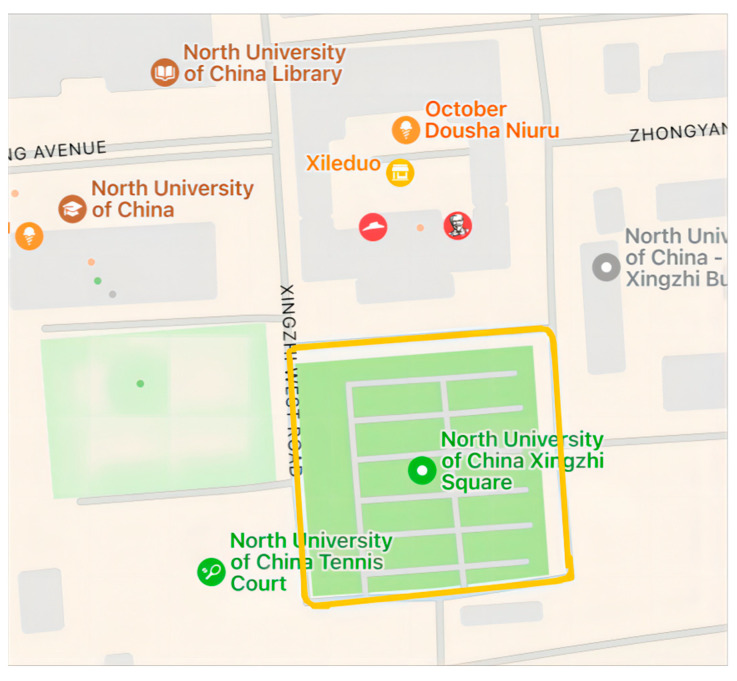
Schematic diagram of precision experiment route.

Starting from Xing Zhi West Road, go around Xing Zhi Square and finally return to the starting point. Figure 13 illustrates the schematic diagram depicting the precision test route.

#### 3.2.2. Stability Tests

**Figure 14:**
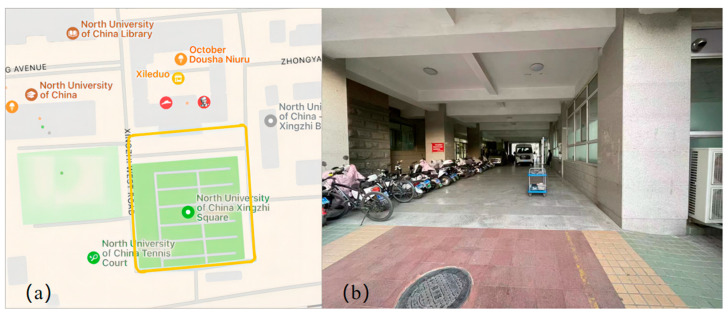
Schematic representation of the stability test course and the complete equipment passing through the tunnel. (**a**) Schematic diagram of stability test route; (**b**) experimental site diagram of the entire equipment passing through the tunnel.

Starting from Xing Zhi West Road, go around Xing Zhi Square pass through the tunnel under the main building of the university, and finally return to the starting point. Figure 14 displays the schematic diagram of the stability test route, including the entire equipment passing through the tunnel site.

## 4. Results and Discussion

### 4.1. Digital Compass Self-Precision Test

**Figure 15:**
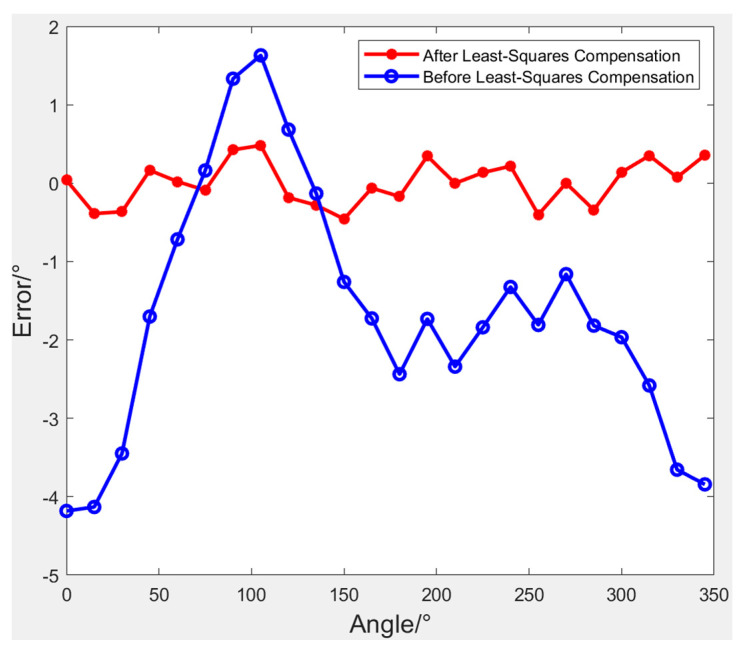
Precision test results of the digital compass itself.

Following numerous measurements, the experimental data underwent various preprocessing procedures, including the removal of outlier terms and adjustments via ellipsoid fitting techniques. The calculated azimuth angle had an error of 4.1824°. After processing the compass error, the error can be significantly reduced. The maximum error value is further reduced to 0.4580°, which is about one order of magnitude lower. The error is significantly reduced by 89.0%. The data before and after least-squares compensation are shown in Table 3. The precision test results of the digital compass itself are shown in Figure 15.

### 4.2. Practical Experiment of Digital Compass in CNS

#### 4.2.1. Precision Experiment

**Figure 16:**
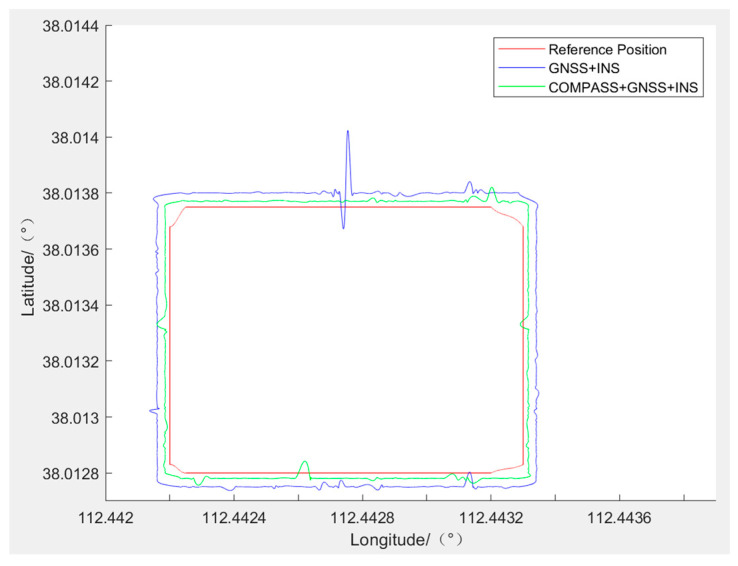
Experiment of the digital compass on integrated navigation precision.

The data collected by the high-precision portable TMR digital compass and the data collected by the GNSS/INS-integrated navigation are processed on MATLAB software. The ultimate outcomes of the experiments are depicted in Figure 16. The outcomes reveal that the red route serves as the reference, with the blue route representing the trajectory produced solely by the GNSS/INS CNS. The green route depicts the path taken by the COMPASS+GNSS/INS CNS. According to the analysis, the route of integrated navigation after adding the high-precision portable TMR digital compass is closer to the reference route than without the high-precision portable TMR digital compass. It can be concluded that the developed high-precision portable TMR digital compass system can improve the overall precision of integrated navigation.

#### 4.2.2. Stability Test

**Figure 17:**
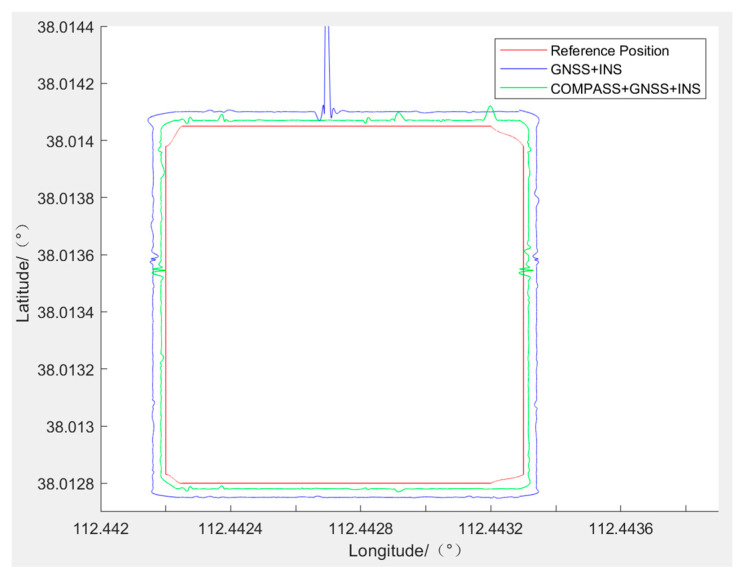
Experiment on the stability of integrated navigation with a digital compass.

The data collected by the high-precision portable TMR digital compass and the data collected by the GNSS/INS integrated navigation are processed on MATLAB software. The conclusive outcomes of the experiments are depicted in Figure 17. The results indicate that the red route serves as the reference path, while the blue route represents the trajectory produced solely by the GNSS/INS CNS. The path depicted in green corresponds to the COMPASS+GNSS/INS CNS route. The examination indicates that with obstacles present (tunnel obstacles in the experiment), the integrated navigation scenario after integrating a high-precision portable TMR digital compass performs superiorly compared to the scenario without the addition of a high-precision portable TMR digital compass. The image noise and mutations are smaller, and the navigation routes are more stable. It can be concluded that the developed high-precision portable TMR digital compass system can improve the stability of integrated navigation.

## 5. Conclusions

This paper mainly performs two functions. First, it developed and designed a high-precision portable tunnel magnetoresistance (abbreviation: TMR) digital compass system. After testing, the diameter of this digital compass system is 4 cm, and the weight is 10 g. It is of a small size, light weight type, thereby fully satisfying portability. In addition, the azimuth precision error of this digital compass before compensation is 4.1824°. The azimuth precision after compensation can reach 0.4580°. The error is reduced by about one order of magnitude, and the error drops significantly by 89.00%. This proves that the developed high-precision portable tunnel magnetoresistance (abbreviation: TMR) digital compass system itself has high performance. The second function is to use the developed high-precision portable tunnel magnetoresistance (abbreviation: TMR) digital compass system in conjunction with the vehicle-mounted Global Navigation Satellite System (abbreviation: GNSS)/Inertial Navigation System (abbreviation: INS) combined navigation system to test the practicality of this digital compass. Following the experiment, it was determined that the developed high-precision portable TMR digital compass system significantly enhances the precision and stability of the tunnel magnetoresistance (abbreviation: TMR) as a whole, which can be widely used in combined navigation systems (abbreviation: CNSs). Because of this, this high-precision portable tunnel magnetoresistance (abbreviation: TMR) digital compass system is suitable for navigation fields such as ship navigation and vehicle navigation.

## Figures and Tables

**Table 1:** Comparison of advantages and disadvantages of four types of magnetoresistance effects.

Category	HALL	AMR	GMR	TMR
Advantage	Low cost CMOS integration	High signal-to-noise ratio	High sensitivity	Extremely high sensitivity
				Large rate of change in magnetic resistance
		High stability		Good linearity
	Large measurement range	Small zero drift		Good temperature stability
		Set/Reset function, automatic calibration of zero point		No interlayer coupling effect
				No need for magnetic ring structure and Set/Reset coils
Disadvantage	Low signal-to-noise ratio	Low range	Large zero drift after being disturbed by a large magnetic field	Large zero drift after being disturbed by a large magnetic field
	Low sensitivity	Narrow linear range	High cost	
	Large zero drift	Complex manufacturing process	Low linear range	
	Only the magnetic field in the Z-axis direction can be measured, and additional magnetic focusing structures are needed to measure signals in the XOY plane, which can result in significant magnetic hysteresis	Set/reset coils need to be set for preset/reset operations	There is interlayer coupling effect, which limit the application	

**Table 2:** Data transmission protocol description.

Description	Header	Header	Data Type	Data Length	Data	XOR Value
Value	0xA5	0x5A	~	~	~	~
Byte Count	1 Byte	1 Byte	1 Byte	1 Byte	(4×n) Byte	1 Byte

**Table 3:** Data before and after least-squares compensation.

Theoretical Angle Value (Unit: °)	Error before Compensation (Unit: °)	Error after Compensation (Unit: °)
0	−4.1824	0.0377
15	−4.132	−0.3884
30	−3.4493	−0.3647
45	−1.702	0.1639
60	−0.7173	0.0198
75	0.1618	−0.0902
90	1.3323	0.4255
105	1.6308	0.4516
120	0.6833	−0.1847
135	−0.1324	−0.2812
150	−1.2602	−0.4580
165	−1.732	−0.0628
180	−2.438	−0.1706
195	−1.732	0.3447
210	−2.341	−0.0018
225	−1.839	0.134
240	−1.325	0.2174
255	−1.809	−0.4019
270	−1.1611	−0.0002
285	−1.818	−0.3419
300	−1.9655	0.1395
315	−2.5814	0.3512
330	−3.6561	0.0749
345	−3.8434	0.3564

## Data Availability

Data are contained within the article.
